# Air in the right ventricle and vein after basilar skull fracture: a case report

**DOI:** 10.1186/s12245-020-00326-5

**Published:** 2020-11-30

**Authors:** Hiroki Kai, Tomoya Hirose, Takaya Nishiura, Takashi Noma, Yoshihito Ogawa, Tomoki Yamada, Haruhiko Nakae, Yasuaki Mizushima

**Affiliations:** Emergency and Critical Care Medical Center, Osaka Police Hospital, 10-31, Kitayama-cho, Tennoji-ku, Osaka-shi, Osaka, 543-0035 Japan

**Keywords:** Basilar skull fracture, Air embolism, Right ventricle, Jugular vein

## Abstract

**Background:**

Air in the venous system may cause vascular air embolism, which is a potentially life-threatening event. The presence of air in venous system after basilar skull fracture is very rare.

**Case presentation:**

A 77-year-old man fell from a truck bed and suffered head and neck trauma. On hospital arrival, his consciousness was clear and his vital signs were stable. His chief complaint was pain in the back of his head and neck. Head CT showed traumatic subarachnoid hemorrhage in the right frontal area and basilar skull fracture of the occipital bone. Whole body CT showed pneumocephalus and air in the jugular vein and right ventricle. The patient was placed in the supine position in a state of absolute rest to prevent vascular air embolism and was treated conservatively. On hospital day 3, CT was reperformed, revealing disappearance of air in the right ventricle and decreased air in the veins of the head and neck. On hospital day 4, the air in the veins disappeared completely on CT. He did not experience vascular air embolism after increasing of his activity level (e.g., raising his head on hospital day 3 and standing and walking alone on day 5). He was discharged 34 days after admission without sequelae.

**Conclusions:**

Head trauma patients with basilar skull fracture might develop vascular air embolism if physicians fail to detect air in the venous system on hospital arrival. A high degree of suspicion regarding air in venous system or heart is required when patients present with such injuries.

## Background

Air in the venous system may result in vascular air embolism that can cause potentially life-threatening events. The occurrence of air in the venous system after basilar skull fracture, which may result in vascular air embolism, is rare [1–3]. To our knowledge, this is the first report of air in the right ventricle after basilar skull fracture in a non-fatal trauma case.

## Case presentation

The patient was a 77-year-old man who fell from the loading platform of a truck (height 1.5 m) and landed on the back of his head. He could initially walk without weakness or other symptoms. However, he gradually developed dyspnea and right hemiplegia and presented to the emergency department of another hospital. The physician found it difficult to diagnose and treat the patient, and he was immediately transferred to our emergency department by ambulance.

On arrival, his consciousness was alert and his vital signs were stable. He felt occipital and posterior cervical pain. There was no dyspnea or right hemiplegia. His medical history included paroxysmal atrial fibrillation, for which warfarin was prescribed. On physical examination, there was no external wound, with only spontaneous pain and tenderness at the back of his head and neck. A motor and sensory examination of the extremities and trunk was normal. After primary and secondary trauma surveys and X-rays of the chest and pelvis, whole body computed tomography (CT) was performed.

**Fig. 1:**
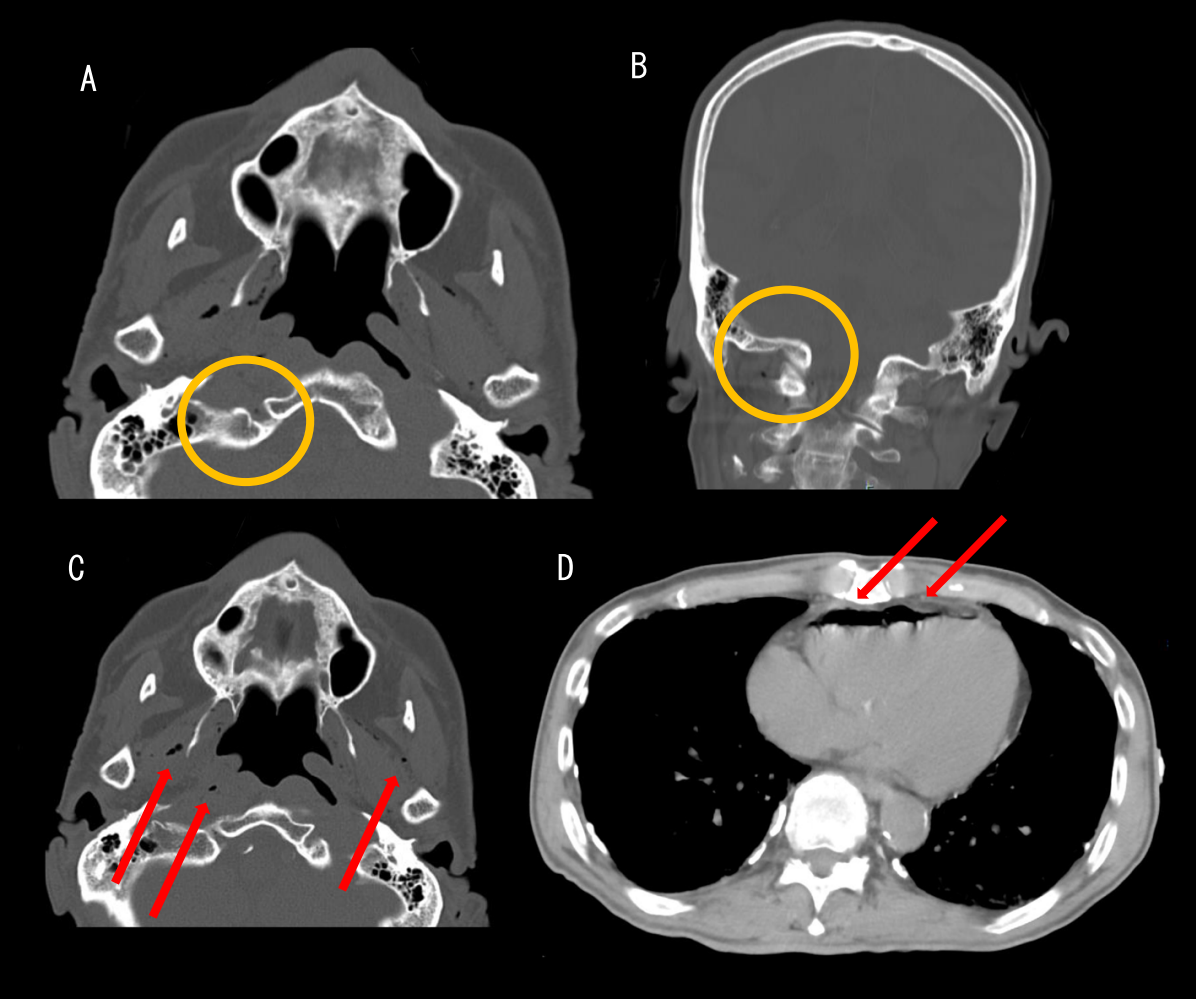
CT scans obtained during trauma evaluation. **a** Head axial CT showed basilar skull fracture of the occipital bone (circle). **b** Head coronal CT showed basilar skull fracture of the occipital bone (circle) as well as **a**. **c** Head axial CT showed air in the vein at the head and neck (arrow). **d** Chest CT showed air in the right ventricle (arrow)

Head CT showed traumatic subarachnoid hemorrhage in the right frontal area and basilar skull fracture of the occipital bone (Fig. 1). Whole body CT showed pneumocephalus and air in the jugular vein and right ventricle. To prevent vascular air embolism, the patient was placed in the supine position in a state of absolute rest after admission and was treated conservatively.

On hospital day 3, head, neck, and chest CT were reperformed, revealing the disappearance of air in the right ventricle and decreased air in the head and neck veins. The patient’s activity level was gradually increased. On hospital day 4, CT was reperformed, revealing the complete disappearance of air in the veins. There were no signs of vascular air embolism, despite an increase in the patient’s activity level, including raising his head on hospital day 3 and standing up and walking alone on hospital day 5. He developed cerebral salt wasting syndrome (CSWS) on hospital day 6. We therefore administered adrenocortical steroids and the patient gradually recovered from hyponatremia. A long period was required to treat hyponatremia. He was discharged on hospital day 34 without sequelae.

## Discussion and conclusions

Basilar skull fracture is a common traumatic injury; however, there are few case reports on basilar skull fracture with air in the veins or heart [1–3]. Anderson and Lube presented a case of jugular venous air after basilar skull fracture in which a 17-year-old boy suffered a basilar skull fracture of the right temporal bone overlying the mastoid air cells in a motor vehicle collision, resulting in pneumocephalus and jugular venous air, as well as multiple punctate hemorrhagic lesions in the brain and acute subdural hematoma [1]. They noted that air embolism can occur due to intracranial air passing through the arachnoid villi or via vessel laceration. Bartynski and Wang reported the case of a 21-year-old man with air in the cavernous sinus with a basilar skull fracture after an acute head injury [2]. Adams and Claude reported several cases of venous air embolism after fatal blunt cranial trauma, in which blunt cranial trauma resulted in open cranial vault and dural vessel laceration [3]. However, in these cases [2, 3], the presence of air resulted from severe trauma or fatal head injury. In our case, the air in the veins and heart might have resulted from a mild basilar skull fracture. In mild trauma cases, physicians may fail to notice air in the venous system if they do not perform chest and neck CT.

Vascular air embolism represents a potentially life-threatening event and must be prevented. Important risk factors for vascular air embolism include surgery and vascular puncture, especially in the sitting position or when the operative site is within 5 cm above the heart [4, 5]. In our case, since the patient had a large amount of air in the right ventricle, we ensured that he remained in a state of absolute rest in the supine position after admission, which kept his head below the level of his heart. Consequently, we could successfully prevent the development of vascular air embolism and the patient was finally discharged without sequelae.

In conclusion, head trauma patients with basilar skull fracture might develop vascular air embolism if physicians fail to notice air in the venous system on hospital arrival. A high degree of suspicion regarding the possible existence of air in venous system is required when patients present with such injuries require.

## Data Availability

Data sharing is not applicable to this article as no datasets were generated or analyzed during the current study.

## Abbreviations

CSWS: Cerebral salt wasting syndrome
CT: Computed tomography
